# Selected Methods for Designing Monetary and Fiscal Targeting Rules Within the Policy Mix Framework

**DOI:** 10.3390/e27101082

**Published:** 2025-10-19

**Authors:** Agnieszka Przybylska-Mazur

**Affiliations:** Department of Statistical and Mathematical Methods in Economics, Faculty of Economics, University of Economics in Katowice, ul. 1 Maja 50, 40-287 Katowice, Poland; agnieszka.przybylska-mazur@ue.katowice.pl

**Keywords:** targeting rules, monetary and fiscal policy mix, dynamic optimization, linear–quadratic regulator, Bellman’s dynamic programming, Euler’s calculus of variations, interest rate, balance-to-GDP ratio, debt-to-GDP ratio

## Abstract

In the existing literature, targeting rules are typically determined separately for monetary and fiscal policy. This article proposes a framework for determining targeting rules that account for the policy mix of both monetary and fiscal policy. The aim of this study is to compare selected optimization methods used to derive targeting rules as solutions to a constrained minimization problem. The constraints are defined by a model that incorporates a monetary and fiscal policy mix. The optimization methods applied include the linear–quadratic regulator, Bellman dynamic programming, and Euler’s calculus of variations. The resulting targeting rules are solutions to a discrete-time optimization problem with a finite horizon and without discounting. In this article, we define targeting rules that take into account the monetary and fiscal policy mix. The derived rules allow for the calculation of optimal values for the interest rate and the balance-to-GDP ratio, which ensure price stability, a stable debt-to-GDP ratio, and the desired GDP growth dynamics. It can be noted that all the optimization methods used yield the same optimal vector of decision variables, and the specific method applied does not affect the form of the targeting rules.

## 1. Introduction

One of the ways to make monetary and fiscal policy decisions is rule-based decision-making. This decision-making approach is particularly significant in achieving established objectives. It should be noted that based on Article 5 of Council Directive (EU) 2024/1265 of 29 April 2024 [1], each Member State shall establish its specific numerical fiscal rules to effectively promote compliance with its obligations deriving from the TFEU in the area of budgetary policy over a multiannual period for the general government as a whole. When making monetary policy decisions, it is also worth basing them on rules that will make it possible to achieve the established monetary policy objective.

Decision-making based on rules, the advantages and consequences of using them, as well as methods for determining rules are the subject of research by many researchers (see [2]). Potrafke [3] presents the economic consequences of fiscal rules in his paper, Darvas et al. [4] present the implications of new fiscal rules in the European Union, while Brzozowski and Siwiska-Gorzelak [5] analyze the impact of fiscal rules on the volatility of fiscal policy.

The primary objective of monetary policy implemented in accordance with the Direct Inflation Targeting Strategy is to achieve a stable price level, while the primary objective of fiscal policy is to achieve stable public finances.

However, the importance of monetary policy rules in achieving price stability and their impact on financial stability has also been analyzed by many researchers (see, e.g., Adrian and Liang [6], Galí [7], Masciandaro [8], and Bernanke et al. [9]).

It should also be noted that a central feature of policy mix studies is the existence of trade-offs between monetary and fiscal policy objectives. In the present framework, these tensions arise naturally from the structure of the model, where price index of consumer goods and services, general government debt-to-GDP ratio and growth rate of GDP evolve as endogenous state variables, while the policy instruments are the interest rate and general government balance-to-GDP ratio.

Policymakers face a multi-objective optimization problem, as the stabilization of inflation and output often conflicts with the need to ensure debt sustainability. For instance, a higher interest rate may help reduce inflationary pressures but simultaneously increases the cost of debt servicing, thereby worsening the fiscal stance. Conversely, a restrictive fiscal policy that improves the primary balance may dampen output growth and further deviate inflation from its target.

These interactions imply that there is no single policy instrument capable of simultaneously achieving all objectives. The optimal policy mix thus depends on the relative weights assigned to price stability, output stabilization, and fiscal sustainability in the policymaker’s loss function subject to the model’s dynamic constraints. The resulting first-order conditions characterize the optimal trade-offs: tighter monetary policy typically lowers inflation at the cost of higher debt ratios, whereas fiscal tightening improves debt dynamics but may amplify output and inflation deviations.

This formal structure allows us to explicitly quantify and discuss the policy trade-offs that lie at the heart of the policy mix debate—particularly the tension between short-term stabilization goals and long-term debt sustainability.

Additionally, when making monetary and fiscal policy decisions, other variables such as, for example, the dynamics of economic growth should also be taken into account. It should be noted that monetary policy and fiscal policy are two different policies, but their interaction is important for achieving the objectives. Moreover, by integrating both policies it is possible to take into account more macroeconomic variables in decision-making than when they are conducted separately.

Although monetary and fiscal policy are conducted separately, their cooperation is needed in the face of new challenges (see, e.g., [10,11,12,13]). Combes et al. [14] analyzed how inflation targeting and fiscal rules affect inflation and fiscal outcomes. They found that integrating inflation targeting and fiscal rules enables more disciplined macroeconomic policy than when monetary and fiscal policy are conducted separately.

The topic is both relevant and original in the context of economic policy optimization. Traditionally, monetary and fiscal policies have been considered separately, and targeting rules are usually defined for each policy in isolation. This research proposes an integrated approach, focusing on the simultaneous interaction between monetary and fiscal policies and offering a comprehensive framework for deriving combined targeting rules. This integrated approach addresses a gap in the field by linking the two policy instruments within a joint optimization framework, offering a more realistic model for policymakers.

The main question addressed by the research is how to derive targeting rules that incorporate both monetary and fiscal policies as part of a policy mix.

In this study, the first verified hypothesis is formulated as follows: To achieve the assumed economic policy objectives, the monetary and fiscal policy mix should be taken into account when making decisions.

One type of rule that can be used in making monetary and fiscal policy decisions is the targeting rule [15].

When making decisions based on targeting rules, it should be noted that in financial and economic problems, the primary objective of optimization is to control the economic system by minimizing deviations of the considered economic and financial variables from their reference values. Generally, control involves influencing a given object in order to achieve the assumed objective. Control is associated with certain information in the form of a signal, while the effect of control is changes in the state of the object. In practice, the control system, which includes the controlled object and the controller, is often affected by external disturbances, whereas the controlled object shows some variability; thus, negative feedback control is of special significance in finance and economics. This is a fundamental self-regulatory mechanism. A self-regulatory mechanism occurs when information about the discrepancy between the actual and reference values of the state variables, i.e., the adopted financial and economic variables, is used to eliminate this difference. Maintaining the values of the state variables at an assumed level is possible when any disturbances causing the deviation of these variables from the reference values, in either direction, induce actions that lead to the elimination or compensation of the effect of these deviations.

Targeting rules are the solution to the problem of minimizing deviations of the considered variables from their reference values under given specific constraints. Most often, targeting rules are a solution to the problem where the objective function is a quadratic function and the constraints are linear (see, e.g., [16,17]). Benigno and Woodford [18] determine optimal monetary and fiscal policy rules based on the problem of maximizing expected discounted utility in a dynamic stochastic general equilibrium model.

The research findings presented in this article extend and complement previous research by using various optimization methods to solve the presented model, as well as by including the model that takes into account the monetary and fiscal policy mix in the optimization problem. In this study, the objectives adopted achieved stable price levels, stable general government debt, and economic growth. In the problem used for analyses, the constraints can be expressed in the form of a state variable equation

The second verified hypothesis was formulated as follows: Optimization methods allow for deriving the solution to the optimal control problem, constituting rules aimed at achieving the objectives of monetary and fiscal policy.

Moreover, targeting rules can be analyzed in the context of information theory, since such policy decisions are made under conditions of limited information available to central banks and governments. Policymakers do not perfectly observe the state of the economy because of external shocks, random disturbances, and imperfect measurement tools. This results in measurement noise—errors arising in the observation or measurement of variables—which can also be caused by delayed data releases or subsequent revisions (e.g., GDP). Consequently, policymakers observe only a noisy approximation of the true state of the economy. Monetary and fiscal policy can therefore be viewed as being conducted under information asymmetry and uncertainty. The targeting rules presented in the paper can be understood as decision rules that act as a decoding function. They transform the encoded and noisy signal, represented by published state variables of the economy—such as the consumer price index for goods and services, the general government debt-to-GDP ratio, and the GDP growth rate—into policy decisions regarding the reference interest rate and the general government balance-to-GDP ratio.

The aim of this article is to compare the solutions obtained from selected optimization methods, such as the linear–quadratic regulator (LQR), Bellman’s dynamic programming, and Euler’s calculus of variations, which are used to derive targeting rules. In this article, we present targeting rules as solutions to an objective function minimization problem with a finite horizon and without discounting, subject to constraints defined by a model that integrates monetary and fiscal policy.

We note that in models where the aim is to examine fiscal–monetary coordination rather than consumer utility maximization, a model without discounting can be used because of the following:

- Decisions are considered from the perspective of authorities making monetary or fiscal policy decisions, who do not have their own time preferences;
- We are interested in the structural equilibrium between policies.

Monetary and fiscal policy can be formulated in a time-neutral manner—assuming equal weighting of today’s and future effects, thereby taking into account so-called intergenerational fairness.

In models that incorporate a policy mix, it can be assumed that the public institutions, such as the government and the central bank, should treat future generations equally with the present one as well as the absence of discounting reflects a normative egalitarian intertemporal approach, which eliminates the arbitrary privileging of the present. This approach is sometimes used in models with a prescriptive or normative character. Therefore, it can be applied in models of optimal fiscal and monetary policy.

It should be noted that, in the context of information theory, targeting rules can be viewed as an optimal signal decoding problem. In this setting, the rule minimizes the sum of squared deviations between the observed variables and the corresponding target values considered in the analysis.

It has been found that various optimization methods can be applied to derive target rules, whereas incorporating a model that accounts for the monetary and fiscal policy mix as a constraint allows for the achievement of all intended objectives, including both monetary and fiscal policy goals.

In the section of the paper presenting the results of empirical analyses, the obtained results for Poland are presented. The results allowed for a comparison of the solutions obtained with various applied methods in the context of achieving the adopted objectives of monetary and fiscal policy.

Therefore, the goal of this manuscript is to apply various methods to solve the proposed optimization problem that incorporates the policy mix. Accordingly, the goal is to advance theoretical modeling and to provide empirical insights that may serve as guidance for monetary and fiscal policy decisions, as well as an incentive for policymakers to consider the inclusion of the policy mix concept—that is, the coordinated approach to fiscal and monetary policy aimed at achieving objectives such as economic growth, price stability, and the sustainability of the general government debt-to-GDP ratio. Existing studies lack theoretical and empirical analyses that take into account the concept of the policy mix and its implications for Poland. This manuscript fills this research gap.

## 2. Methods and Materials

In this section, we present the theoretical foundations of the optimization problem and the methods used to solve it. Next, we present the materials used in the empirical analysis conducted for Poland.

### 2.1. Methods

At the beginning, we presented the model that incorporates the monetary and fiscal policy mix. This presented model includes both monetary and fiscal policy decision variables. Moreover, this model, which represents the constraints in the optimization problem, can be written in the form state variable model.

Next, the theoretical foundations of the methods used to solve the discrete optimization problem are presented:

(1) Linear–quadratic regulator (LQR).
(2) Selected methods of direct dynamic optimization:
  - Bellman’s optimality principle for the problem without discounting and for the discrete case;
  - Euler’s discrete calculus of variations.

The use of the linear–quadratic regulator (LQR) as one of the methods for solving the proposed optimization model required the assumption of a linear constraint condition. The linear model is composed of equations that are frequently used in the analysis of monetary and fiscal policy.

The LQR method is a classical optimization approach for linear dynamic systems with a quadratic cost function. This method is analytical, computationally efficient, and often used as a reference benchmark. However, it requires the assumption of linear constraints and a quadratic form of the cost function.

As alternative methods for solving the proposed optimization model, such as Bellman’s optimality principle for the problem without discounting and in the discrete case as well as the Euler method of discrete calculus of variations were applied. The applied Bellman’s optimality principle also guarantees an optimal solution for the discrete problem without discounting. It should be noted that the chosen discrete Euler method of variational calculus is intuitive and relatively straightforward to implement.

In this article, the use of Euler’s calculus of variations and Bellman’s dynamic programming requires the adoption of the linearity assumptions of the function f in the state equation in the general model.

This assumption may limit the applicability of the model to real-world economies with more complex behaviors. However, this does not limit the applicability of the selected optimization methods to solving models that form the basis for deriving policy rules incorporating the new Keynesian Phillips curve, the equation describing the dynamics of the government debt-to-GDP ratio, and a modified IS curve capturing the impact of monetary policy decisions on GDP growth.

#### 2.1.1. Monetary and Fiscal Policy Mix Model

In the model, which can be written as a state vector equation, we take into account both monetary policy decision variable concerning the primary monetary policy instrument, i.e., the interest rate, and fiscal policy decision variable regarding the general government deficit-to-GDP ratio.

Considering the conduct of monetary policy and the strategy of direct inflation targeting, the impact of the interest rate decision on inflation is significant. Thus, the first equation of the model represents the new Keynesian Phillips curve with the nominal interest rate, which is a key element of modern macroeconomic models, particularly in the context of monetary policy. It should be noted that many variants of the new Keynesian Phillips curve can be found in the economic literature.

Taking into account the use of the presented model and the empirical context, the curve form presented in the model synthesizes approaches described in the macroeconomic literature on new Keynesian models of inflation and monetary policy (see, e.g., [19,20,21,22,23]). It shows the relationship between the inflation rate, the inflation rate in the previous period, and economic growth in the previous period and the nominal interest rate.

The fiscal component in the model is introduced by incorporating the dynamics of the general government debt-to-GDP ratio, based on its lagged value and the general government balance-to-GDP ratio, instead of relying on the standard budget constraint equation that explicitly accounts for the debt service channel dependent on the interest rate. The rationale for this modeling choice is that the interest rate is treated as one of the model’s instruments determined within the policy mix framework, rather than as a fixed instrument considered only when making fiscal policy decisions.

In the equation used in the model to describe the dynamics of deviations of the general government debt-to-GDP ratio from its target, the parameters $b_{1}$ and $b_{2}$ can be interpreted as follows:

- Parameter $b_{1}$: the deviation of the general government debt-to-GDP ratio from its target in period t changes by $b_{1}$ units (i.e., $b_{1}\cdot 100$ percent) if the deviation of the general government debt-to-GDP ratio from its target in period t−1 increases by 0.01 (i.e., by 1%), ceteris paribus, meaning for an unchanged deviation of the general government balance-to-GDP ratio;
- Parameter $b_{2}$: the deviation of the general government debt-to-GDP ratio from its target in period t changes by $b_{2}$ units (i.e., $b_{2}\cdot 100$ percent) if the deviation of the general government balance-to-GDP ratio from its target in period t increases by 0.01 (i.e., by 1%), ceteris paribus, meaning for an unchanged deviation of the general government debt-to-GDP ratio from its target in period t−1.

This approach allows the fiscal authority to assess the expected change in the deviation of the general government debt-to-GDP ratio from its target in the next period, conditional on the current deviation of the debt ratio from less the target and the contemporaneous deviation of the general government balance-to-GDP ratio from its target. Such a framework is particularly useful for budget planning in the subsequent period.

The third equation in the model additionally includes the impact of monetary policy decisions on the growth rate of GDP. This is related to the fact that when making decisions regarding the interest rate, in addition to a stable price level, GDP dynamics may also be taken into account as an additional, but significant objective. The presented relationship is a modification of the IS curve. It is necessary to emphasize the impact of this relationship on economic equilibrium, as well as the significant importance of the IS curve in macroeconomic analysis, particularly in the context of fiscal and monetary policy.

In order to minimize deviations from target values, the study employed a version of the model that accounts for deviations of individual variables from their targets.

Therefore, we used a model that takes into account both monetary and fiscal policy decisions in the following form:

(1) $$E_{t-1}\pi_{t}-\pi^{*}=a_{1}\left(\pi_{t-1}-\pi^{*}\right)+a_{2}\left(y_{t-1}-y^{*}\right)+a_{3}{(i}_{t}-i^{*})$$

(2) $$d_{t}-d^{*}=b_{1}(d_{t-1}-d^{*})\;{-b}_{2}(s_{t}-s^{*})$$

(3) $$y_{t}{-y}^{*}=c_{1}(\pi_{t-1}{-\pi }^{*})+\;c_{2}(y_{t-1}-y^{*})-c_{3}(i_{t-1}-i^{*})$$

where $i_{t}$—interest rate in period *t*; $E_{t-1}\pi_{t}$—expected price index of consumer goods and services in period *t*, based on period *t* − 1; $\pi_{t-1}$—price index of consumer goods and services in period *t* − 1, $\pi_{t-1}=\frac{P_{t-1}}{P_{t-2}}$; $P_{t-1}$—price level in period t−1; $P_{t-2}$—price level in period t−2, $d_{t}=\frac{D_{t}}{Y_{t}}$—general government debt-to-GDP ratio in period t; $D_{t}$—general government debt in period t; $s_{t}=\frac{S_{t}}{Y_{t}}-$ general government balance-to-GDP ratio in period *t*; $S_{t}-$ general government balance in period t; $Y_{t}$—GDP in period t; $d_{t-1}=\frac{D_{t-1}}{Y_{t-1}}$—general government debt-to-GDP ratio in period t−1; $D_{t-1}$—general government debt in period t−1; $Y_{t-1}$—GDP in period t−1; $y_{t-1}=\frac{Y_{t-1}}{Y_{t-2}}$—growth rate of GDP in period t−1; $\pi^{*},\;y^{*},\;d^{*},\;i^{*},\;s^{*}$—constant values of the inflation target, growth rate of GDP, general government debt-to-GDP ratio, interest rate, general government balance-to-GDP ratio, respectively; and $a_{1},a_{2},\;a_{3},\;b_{1},b_{2}$—model parameters.

It should be noted that one of the approaches to the expected price index of consumer goods and services applied in this paper is the use of the rational expectations approximation. In practice, expectations are not fully rational; however, in many models, rational expectations constitute a standard assumption that simplifies the analysis and ensures internal consistency. An alternative approach is to rely on survey data to estimate the expected price index of consumer goods and services

The above model can be written as a state space model in the following form:

(4) $$x_{t}=Ax_{t-1}+Bu_{t}$$

where $x_{t}$—state variable vector in period *t*; $x_{t}=\left[\begin{array}{c} E_{t-1}\pi_{t}-\pi^{*}\\ d_{t}-d^{*}\\ y_{t}-y^{*} \end{array}\right]$; $x_{t-1}$—state variable vector in period t−1; $x_{t-1}=\left[\begin{array}{c} \pi_{t-1}-\pi^{*}\\ d_{t-1}-d^{*}\\ y_{t-1}-y^{*} \end{array}\right]$; $u_{t}$—vector of decision variables and vector of control variables in period *t*; $u_{t}=\left[\begin{array}{c} i_{t}-i^{*}\\ s_{t}-s^{*} \end{array}\right]$; and A, B—parameter matrices in the constraints. *A*$=\left[\begin{array}{ccc} \alpha_{1} & \alpha_{2} & 0\\ 0 & 0 & b_{1}\\ c_{1} & 0 & c_{2} \end{array}\right]$,$\;B=\left[\begin{array}{cc} \alpha_{3} & 0\\ 0 & -b_{2}\\ -c_{3} & 0 \end{array}\right]$.

We assumed a quadratic form of the objective function and a finite time horizon. Thus:

(5) $$J=\sum_{t=1}^{N}[w_{\pi }{\left(\pi_{t}-\pi^{*}\right)}^{2}+w_{y}{\left(y_{t}-y^{*}\right)}^{2}+w_{d}{\left(d_{t}-d^{*}\right)}^{2}+w_{t}{\left(i_{t}-i^{*}\right)}^{2}+w_{s}{\left(s_{t}-s^{*}\right)}^{2}]$$

Therefore, the problem without discounting and with a finite time horizon considered in the article can be written as follows:

(6) $$\sum_{t=1}^{N}\begin{array}{c} {}[w_{\pi }{\left(\pi_{t}-\pi^{*}\right)}^{2}+w_{y}{\left(y_{t}-y^{*}\right)}^{2}+w_{d}{\left(d_{t}-d^{*}\right)}^{2} \end{array}\;\;+w_{t}{\left(i_{t}-i^{*}\right)}^{2}+w_{s}{\left(s_{t}-s^{*}\right)}^{2}]\;\to min$$

subject to the constraints of the equation system (4) and assuming that the initial condition for t=0 is given, $x_{0}=\left[\begin{array}{c} \begin{array}{c} \pi_{0}-\pi^{*}\\ d_{0}-d^{*} \end{array}\\ y_{0}-y^{*} \end{array}\right]$, where $\pi_{0},\;d_{0},\;y_{0}$ are the initial values of inflation, the initial values of general government debt-to-GDP ratio, and the initial values of growth rate of GDP, respectively.

In the problem considered in this article we assumed a free value of the state variable in the final period, i.e., we considered a case in which $x_{T}$ is free.

The theoretical foundations of the methods used in this article to solve the presented model are presented below.

#### 2.1.2. Linear–Quadratic Regulator (LQR) Method

Since in the above model, the objective function is quadratic and the constraints are linear, the linear–quadratic regulator (LQR) can be applied to solve such a problem. When solving a linear–quadratic problem, we should minimize the cost function, i.e., minimize the deviations of variables from their reference values subject to given constraints.

The regulator, based on control signals and taking into account the predetermined values of the variables characterizing the economy, controls the economy so that it behaves in the desired manner.

When we consider a discrete time approach, the dynamic system representing the constraints in the linear–quadratic problem is the system of the linear difference equations, while the cost function, which is the objective function, is the sum of the squares of the state variables and control variables, i.e., the deviations of the variables from their reference values at each periods. The parameters of the objective function are weights determined by the decision maker. The necessity of specifying these weights is a significant drawback in this method.

It should be noted that the linear–quadratic regulator (LQR) can be used to solve problems with a finite time horizon as well as those with an infinite time horizon.

The problem (6) presented above with the constraints (4) is an example of a finite-horizon discrete-time optimal control problem, i.e., in cases where there is a fixed period in which the decision maker must achieve the assumed objectives.

Therefore, the considered problem can be expressed in a general form as follows:

(7) $$\left\{\begin{array}{c} J=\sum_{t=1}^{N}\left(x_{t}^{T}Qx_{t}+u_{t}^{T}Ru_{t}\right)\to min\\ x_{t}=Ax_{t-1}+Bu_{t} \end{array}\right.\;$$

where $x_{t}$ is the vector of variables characterizing the economy and represents the state variables in period *t*; $u_{t}$ is the vector of decision variables and representsthe control variables in period *t*; Q and R are the weight matrices in the objective function; A, and B are the parameter matrices in the constraints.

The solution to the above problem is calculated using the formula:

(8) $$u_{t}=-F_{t+1}x_{t}\mathrm{for}\;t=0,\;1,\;2,\;\ldots ,\;n-1$$

where

(9) $$F_{t}={\left(R+B^{T}P_{t}B\right)}^{-1}B^{T}P_{t}A$$

and $P_{t}$ is determined by solving the dynamic Riccati equation using the backward iteration method:

(10) $$P_{t-1}={Q+A^{T}(P_{t}-P_{t}B\left(R+B^{T}P_{t}B\right)}^{-1}B^{T}P_{t})A$$

with initial condition $P_{N}=Q$.

#### 2.1.3. Bellman’s Dynamic Programming Method

To solve the presented problem (6) with the constraints (4), which represents a model incorporating the monetary and fiscal policy mix, dynamic optimization methods can also be used. One of the dynamic optimization methods used in the article is Bellman’s dynamic programming. Unlike the linear–quadratic regulator (LQR), the Bellman dynamic programming method can be applied not only to linear–quadratic problems but also to optimal control problems that may be nonlinear and stochastic.

In the problem, based on which we derive the targeting rule, the analytical form of the function in the state equation is the same in all periods, and also the analytical form of the objective function is the same in all periods. This is an example of an optimal control problem without discounting in the discrete case in a finite time horizon. Assuming the minimization of the objective function, we can formulate this optimal control problem in general form as follows:

(11) $$\left\{\begin{array}{c} J=\sum_{t=1}^{N}f^{0}(x_{t},u_{t})\to min\\ x_{t}=f(x_{t-1},u_{t}) \end{array}\right.\;$$

where $x_{t}$ is the vector of variables characterizing the economy and represents the state variables in period *t*; $x_{t-1}$ is the vector of variables characterizing the economy and represents the state variables in period *t* − 1; $u_{t}$ is the vector of decision variables and represents the control variables in period *t*; $f^{0}\left(x,u\right)$ is the function characterizing the efficiency of the chosen control; $f\left(x,\;u\right)\;$ is the function describing the law of motion of a discrete controlled object and constitutes the constraints of the problem. This function is a vector function and takes values in space $R^{n}$.

We note that in the fundamental optimal control problem, the initial value $x_{0}$ of the state variable vector should be assumed.

When we consider the fundamental problem, knowing the initial value $x_{0}$ of the state variable vector, the goal of optimal control is to select the controls $u_{1},\;u_{2},\;\ldots ,\;u_{N},$ such that $u_{t}\in U_{t}\left(x_{t-1}\right)$ for t=1, 2, …, N satisfying the equation $x_{t}=f(x_{t-1},u_{t})$ and the condition $\sum_{t=1}^{N}f^{0}(x_{t},u_{t})\to min$. Moreover, the obtained sequence ${x_{0},\;x}_{1},\;x_{2},\;\ldots ,\;x_{N}$ of the states in periods t=0, 1, 2, …, N is called the trajectory.

Assuming $x_{t}=x$, $J_{k}=\;\sum_{t=k}^{N}f^{0}(x_{t},u_{t})$, and $M_{k}\left(x\right)=\min\sum_{t=k}^{N}f^{0}\left(x_{t},u_{t}\right),$ the discrete solution of the optimality principle for problem (11), known as Bellman’s equation, is given by:

(12) $$\begin{array}{c} M_{k-1}\left(x\right)=\min\left\{f^{0}(x,u\right)+M_{k}(x)\}\;for\;k=2,3,\;\ldots ,\;N\\ u\in U_{k}\left(x\right)\; \end{array}$$

This allows for the sequential calculation of functions $M_{N}\left(x\right),\;M_{N-1}\left(x\right),\;\ldots .\;M_{2}\left(x\right),\;M_{1}\left(x\right)$ starting from $M_{N}\left(x\right)=0$, thereby sequentially determining the controls $u_{t}$ and states $x_{t}$. Additionally, $M_{1}\left(x\right)=\min\sum_{t=1}^{N}f^{0}(x_{t},u_{t})$.

#### 2.1.4. Method of the Discrete Euler’s Calculus of Variations

Euler’s calculus of variations, similar to Bellman’s dynamic programming method, can be applied not only to the linear–quadratic problem but also to solve problems in which the objective function $f^{0}$ and the function f in the state equation can have any form, unlike the linear–quadratic regulator (LQR).

Since problem (6) with constraints (4) is an optimal control problem for a discrete time with a finite time horizon and with the predetermined initial condition and with a free terminal condition, the foundations of theory concerning the discrete Euler’s calculus of variations are given below.

Analogous to the problem solved using Bellman’s dynamic programming method, the control object is described in the general case by a difference state equation in the following form, $x_{t}=f\left(x_{t-1},u_{t}\right)$. Assuming that there is the predetermined initial state $x_{0}$ and a finite time horizon *N*, it is necessary to determine the control $u_{t}$ for t=1, 2, …, N that minimizes the objective function J and it is necessary to determine the corresponding optimal trajectory $x_{t}$ for t=0, 1, 2, …, N. Thus, the problem (6) with constraints (4) is an example of problem (11).

The necessary condition for the existence of an extremum of the objective function J is that the first variation is zero.

(13) δJ=0

Using the definition of the first variation, the necessary condition for the existence of a local extremum can also be written as the discrete Euler–Lagrange equation in the following form:

(14) $$\frac{\partial f^{0}(,x_{t},u_{t})}{\partial x_{t}}\;+\;\frac{\partial f^{0}(,x_{t-1},u_{t-1})}{\partial x_{t}}\cdot \frac{\partial f(x_{t-1},u_{t-1})}{\partial x_{t-1}}=0\;\mathrm{for}\;\mathrm{all}\;t=2,\;3,\;\ldots ,N$$

To verify whether the control, which is the solution of Equation (14), is the optimal control, i.e., the targeting rule for which the function J reaches its minimum, we need to verify the sufficient condition, according to which the second variation should be positive.

(15) $$\delta^{2}J>0$$

Note that the second variation for the discrete case is calculated from the following formula:

$$\begin{array}{c} \delta^{2}J=\sum_{t=1}^{N}\left({\delta x_{t}}^{T}\frac{{\;\partial^{2}f}^{0}\left(x_{t},u_{t}\right)}{\partial x_{t}^{2}}\delta x_{t}+2{\delta x_{t}}^{T}\frac{{\partial^{2}\;f}^{0}\left(x_{t},u_{t}\right)}{\partial x_{t}\partial u_{t}}\delta u_{t}+{\delta u_{t}}^{T}\frac{{\partial^{2}f}^{0}\left(x_{t},u_{t}\right)}{\partial u_{t}^{2}}\delta u_{t}\right)=\\ \;\;=\sum_{t=1}^{N}{\left[\begin{array}{c} \delta x_{t}\\ \delta u_{t} \end{array}\right]}^{T}H_{t}\left[\begin{array}{c} \delta x_{t}\\ \delta u_{t} \end{array}\right] \end{array}$$

where $H_{t}=\left[\begin{array}{cc} \frac{{\;\partial^{2}f}^{0}\left(x_{t},u_{t}\right)}{\partial x_{t}^{2}} & \frac{{\partial^{2}\;f}^{0}\left(x_{t},u_{t}\right)}{\partial x_{t}\partial u_{t}}\\ \frac{{\partial^{2}\;f}^{0}\left(x_{t},u_{t}\right)}{\partial x_{t}\partial u_{t}} & \frac{{\partial^{2}f}^{0}\left(x_{t},u_{t}\right)}{\partial u_{t}^{2}} \end{array}\right]$ is the Hessian matrix of the function $f^{0}\left(x_{t},u_{t}\right)$ and $\delta x_{t},\;\delta u_{t}$ denote the perturbations of the variables in the state and control vectors, respectively. Therefore, we need to verify the negative definiteness of the Hessian matrices $H_{t}$. Additionally, when we verify the definiteness of the Hessian matrix $H_{t+1}$, we must take into account the state space equation.

### 2.2. Materials

We conducted the empirical analysis using quarterly data for Poland from Q3 2010 to Q1 2024. The dataset includes the following:

- Reference interest rate—end-of-quarter data (source: [24]).
- General government balance-to-GDP ratio—from Q3 2010 to Q2 2012 based on ESA 95, and from Q3 2012 to Q1 2024 based on ESA 2010 (source: [25]).
- Quarterly consumer price index for goods and services (source: [26]).
- General government debt-to-GDP ratio (source: [27]).
- Gross Domestic Product indices—seasonally unadjusted, in annual constant prices of the previous year (source: [25]).

The target values adopted for the analysis were as follows:

- Price index of consumer goods and services: $\pi^{*}=1.025$.
- GDP growth rate: $y^{*}=0.031$—the target for 2024 as stated in the 2024 Budget Act.
- General government debt-to-GDP ratio: $d^{*}=0.55$—the second prudential threshold.
- Reference interest rate: $i^{*}=0.045$—the forecast for Q2 2024, determined from the trend using a 6th-degree polynomial.
- General government balance-to-GDP ratio: $s^{*}=-0.03$—corresponding to a deficit of 3% of GDP, as defined by the convergence criteria.

The following programs were used for the empirical analysis: Gretl (https://gretl.sourceforge.net/, accessed on 30 March 2025), MS Excel, and MatLab (https://ww2.mathworks.cn/products/matlab.html, accessed on 30 March 2025).

Using the Matlab software, the solution of Bellman’s dynamic programming method was obtained. The Matlab code for solving the optimization problem is provided in the Appendix A. The linear–quadratic regulator (LQR) computation was carried out using MS Excel.

## 3. Results and Discussion

Based on the actual values of the variables and their respective target values, we calculated the control and state variables as deviations from their targets. These deviations we used to estimate the model parameters.

The estimated model (1)–(3) is in the following form:

$$E_{t-1}\pi_{t}-1.025=0.9612212\left(\pi_{t-1}-1.025\right)+0.222054\left(y_{t-1}-0.031\;\right)--0.0431{(i}_{t}-0.045)$$

$$d_{t}-0.55=0.9102691\left(d_{t-1}-0.55\right)+0.0070764(s_{t}+0.03)$$

$$y_{t}-0.031=0.072456\left(\pi_{t-1}-1.025\right)+\;0.5819569\left(y_{t-1}-0.031\;\right)--0.17166(i_{t-1}-0.045)$$

We assume that all state variables—namely, the price index of consumer goods and services, the general government debt-to-GDP ratio, and the GDP growth rate—are equally important. Furthermore, we assume that monetary policy and fiscal policy represent two coequal components within the considered policy mix framework, which implies assigning equal weights to the monetary and fiscal policy instruments.

Therefore, we assume equal weights for all state variables in the objective function equal to 1/3, as well as equal weights for monetary and fiscal policy instruments in the objective function equal to 1/2.

Thus, the estimated optimalisation model can be written as follows:

$$\sum_{t=1}^{N}\left[\frac{1}{3}{\left(\pi_{t}-1.025\right)}^{2}+\frac{1}{3}{\left(y_{t}-0.031\right)}^{2}+\frac{1}{3}{\left(d_{t}-0,55\right)}^{2}+\frac{1}{2}{\left(i_{t}-0.045\right)}^{2}+\frac{1}{2}{\left(s_{t}+0.03\right)}^{2}\right]\to \;\to min$$

$$E_{t-1}\pi_{t}-1.025=0.9612212\left(\pi_{t-1}-1.025\right)+0.222054\left(y_{t-1}-0.031\right)-\;-0.0431{(i}_{t}-0.045)\;\;d_{t}-0.55=0.9102691\left(d_{t-1}-0.55\right)+0.0070764(s_{t}+0.03)\;\;y_{t}-0.031=0.072456\left(\pi_{t-1}-1.025\right)+\;0.5819569\left(y_{t-1}-0.031\;\right)-\;-0.17166(i_{t-1}-0.045)$$

Therefore, the resulting matrices were as follows:

$$A=\left[\begin{array}{ccc} 0.9612212 & 0.222054 & 0\\ 0 & 0 & 0.9102691\\ 0.072456 & 0 & 0.5819569 \end{array}\right],\;B=\left[\begin{array}{cc} -0.0431 & 0\\ 0 & 0.0070764\\ -0.17166 & 0 \end{array}\right]$$

and the weighting matrices in the objective function were $Q=\left[\begin{array}{ccc} \frac{1}{3} & 0 & 0\\ 0 & \frac{1}{3} & 0\\ 0 & 0 & \frac{1}{3} \end{array}\right]$, $R=\left[\begin{array}{cc} \frac{1}{2} & 0\\ 0 & \frac{1}{2} \end{array}\right]$.

The initial state vector was $x_{0}=\left[\begin{array}{c} -0.003\\ -0.007\\ 0.008 \end{array}\right]$, reflecting the deviations of the state variables from their targets in Q3 2010.

The solutions of the model were obtained using the methods described in the theoretical section. Specifically, we calculated the LQR and the solution using Bellman’s dynamic programming method and applied the discrete Euler calculus of variations. These methods yielded the optimal deviations of the control variables and state variables from their target values. Table 1, Table 2 and Table 3 present the obtained optimal deviation values.

**Figure 1:**
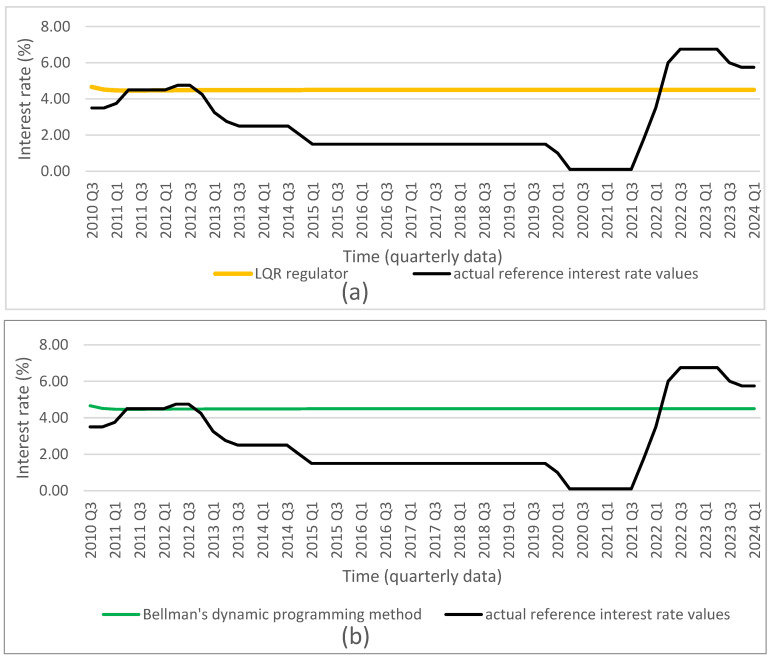

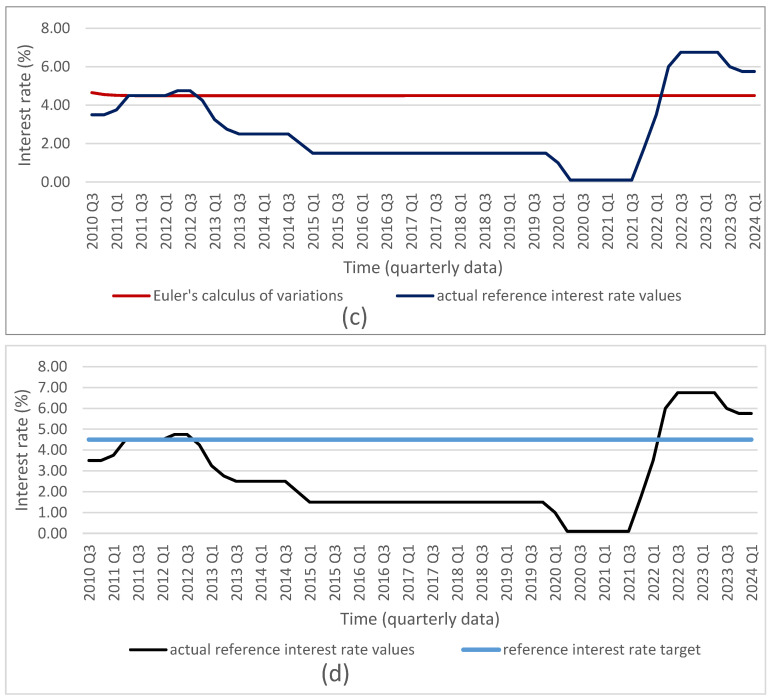
Comparison of actual reference interest rate values (source: NBP) with results from (**a**) LQR (own calculations); (**b**) Bellman’s dynamic programming method (own calculations); (**c**) Euler’s calculus of variations (own calculations); and (**d**) target values (source: NBP).

**Figure 2:**
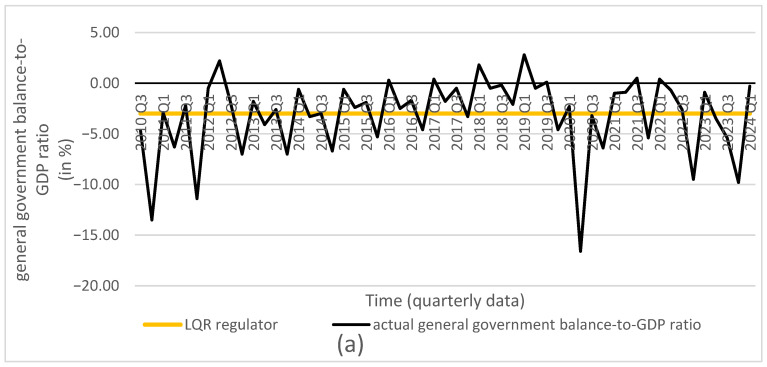

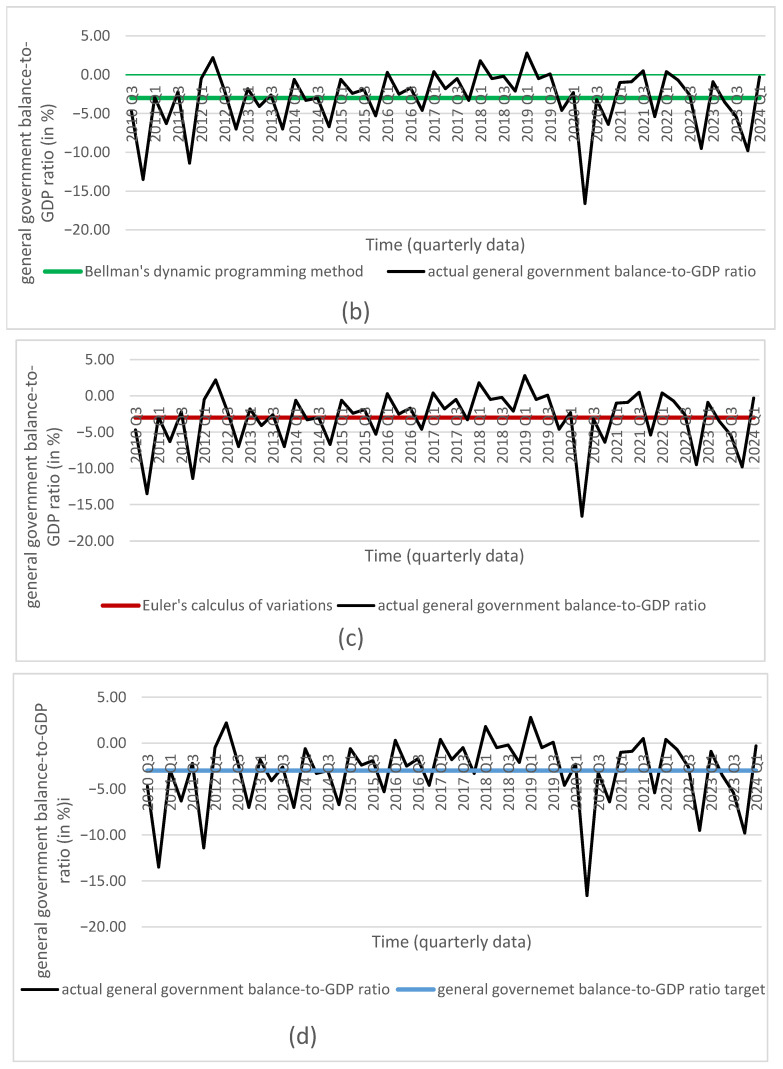
Comparison of actual general government balance-to-GDP ratio (source: Central Statistical Office of Poland) with results from (**a**) LQR (own calculations); (**b**) Bellman’s dynamic programming method (own calculations); (**c**) Euler’s calculus of variations (own calculations); and (**d**) target values (source: Central Statistical Office of Poland).

**Figure 3:**
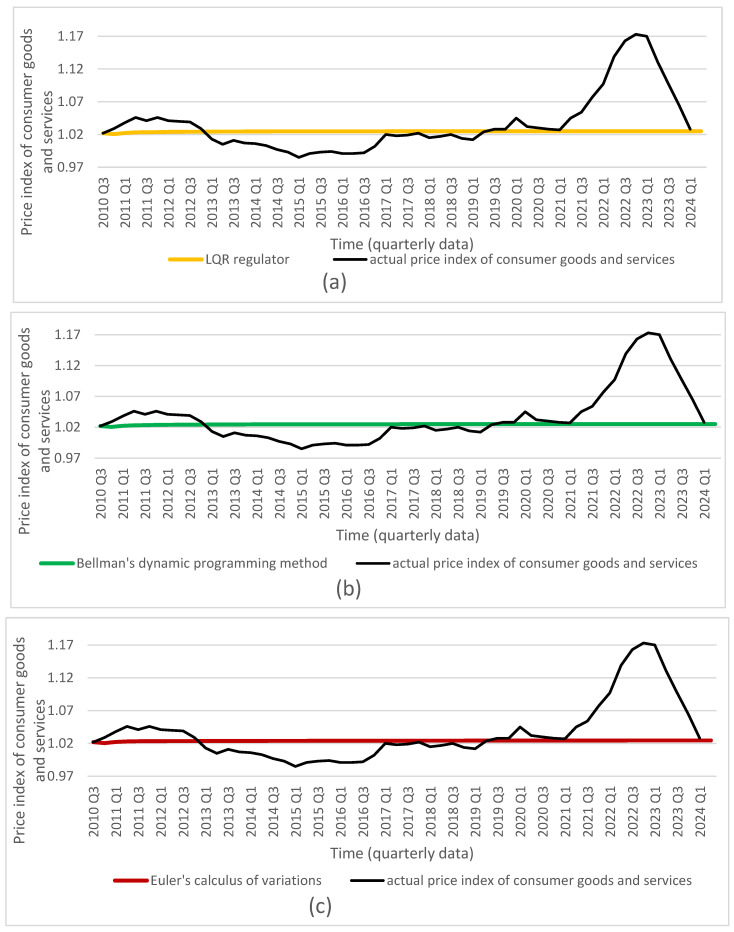

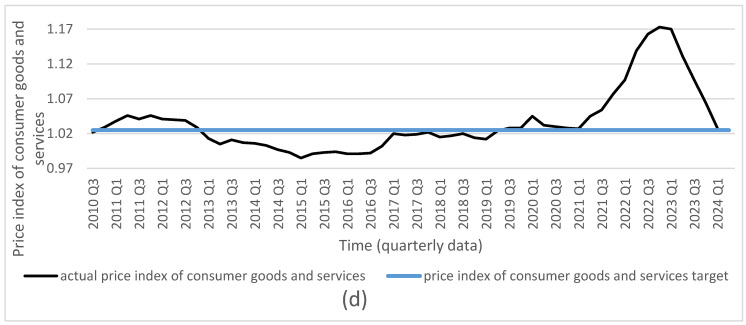
Comparison of actual price index of consumer goods and services (source: Central Statistical Office of Poland) with results from (**a**) LQR (own calculations); (**b**) Bellman’s dynamic programming method (own calculations); (**c**) Euler’s calculus of variations (own calculations); and (**d**) target values (source: Central Statistical Office of Poland).

**Figure 4:**
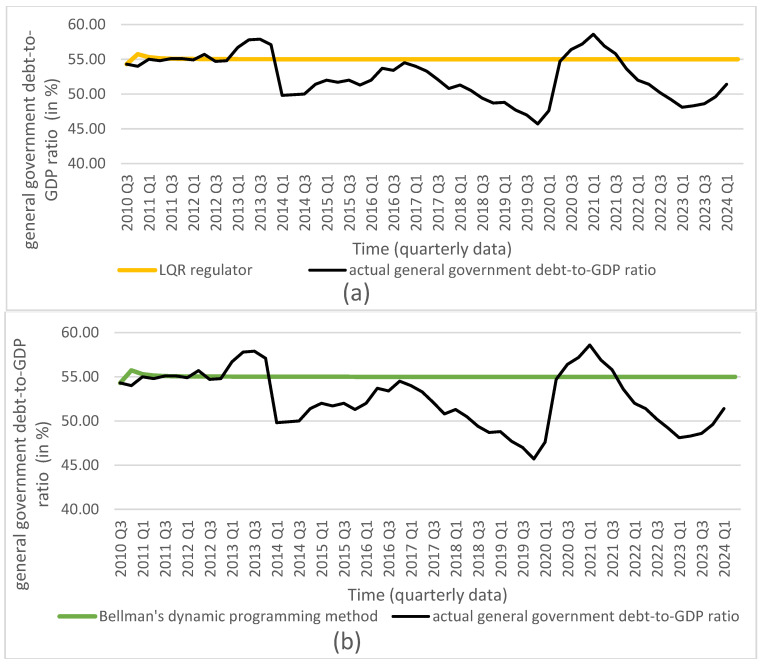

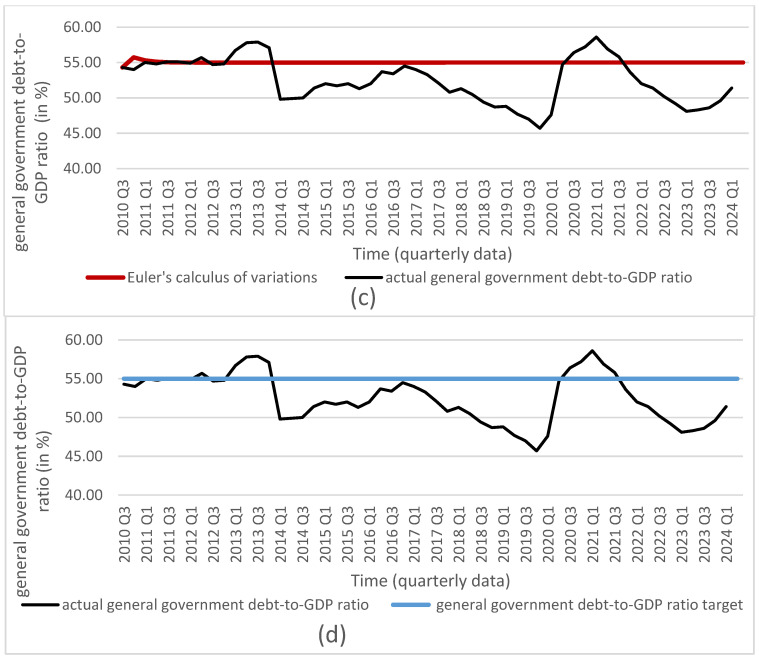
Comparison of general government debt-to-GDP ratio (source: Central Statistical Office of Poland) with results from (**a**) LQR (own calculations); (**b**) Bellman’s dynamic programming method (own calculations); (**c**) Euler’s calculus of variations (own calculations); and (**d**) target values (source: Central Statistical Office of Poland).

**Figure 5:**
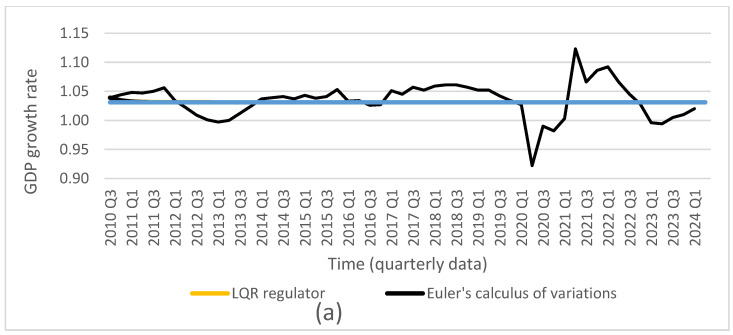

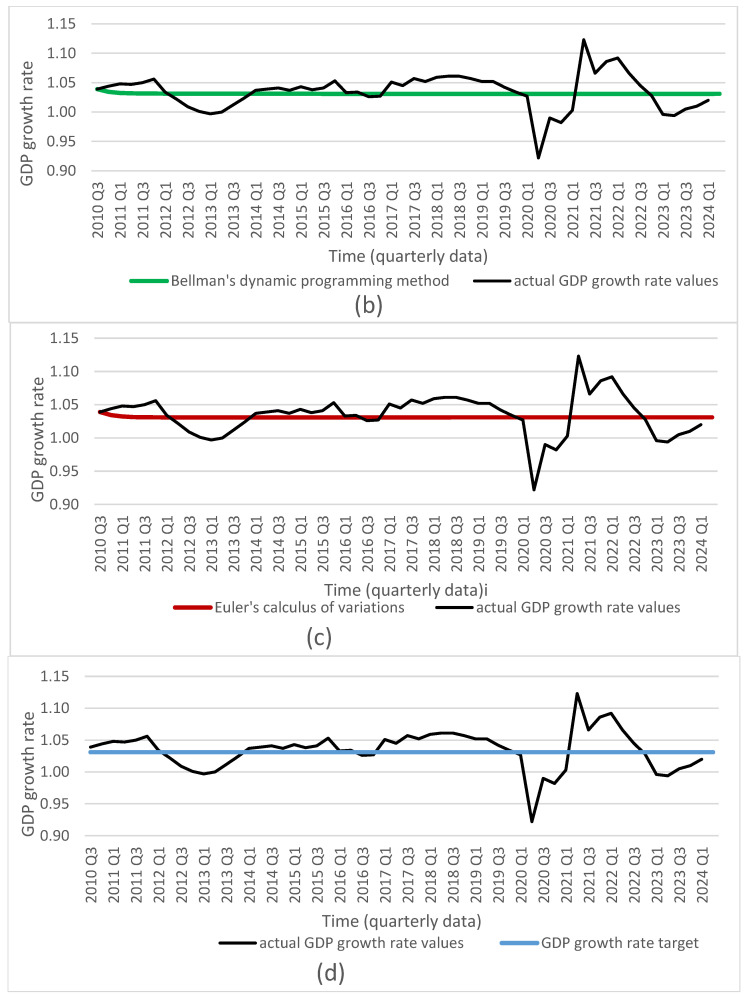
Comparison of GDP growth rate: (source: Central Statistical Office of Poland) with results from (**a**) LQR (own calculations); (**b**) Bellman’s dynamic programming method (own calculations); (**c**) Euler’s calculus of variations (own calculations); and (**d**) target values (source: Central Statistical Office of Poland).

Based on the obtained optimal solutions and the assigned target values, we calculated the optimal levels of the individual control and state variables, which are presented in Figure 1, Figure 2, Figure 3, Figure 4 and Figure 5. The optimal values and target values were then compared with the actual values.

It should be noted that the proposed optimization model can be solved using different methods. All employed methods yield consistent solutions that converge to the target values for all considered decisions and state variables.

Based on the obtained results, we conclude that all dynamic optimization methods applied in the study—such as the LQR, Bellman’s dynamic programming, and Euler’s calculus of variations—used for the discrete linear–quadratic problem with a finite time horizon (without discounting), an assumed initial condition, and a free terminal condition, yield equivalent optimal solutions for the decision variables: the reference interest rate and the general government balance-to-GDP ratio. These optimal solutions achieve the assumed goal, namely minimizing the deviations of all variables, and indicate the optimal trajectories of the state variables: the consumer price index, the general government debt-to-GDP ratio, and the GDP growth rate. This finding supports the second hypothesis.

Furthermore, if the interest rate and the general government balance-to-GDP ratio are set to the calculated optimal values in decision-making, it is possible to achieve the assumed targets for the state variables mentioned above. Therefore, we conclude that using the solutions obtained with any of the presented optimization methods leads to convergence of the state variables to their target values. Moreover, the proposed model, which takes into account both monetary and fiscal policy decisions, enables the achievement of the targeted goals for all state variables that are relevant to the implementation of monetary or fiscal policy.

The solutions presented in this paper—formulated as targeting rules—allow for the monetary and fiscal policy mix. They show that achieving the objectives of monetary and fiscal policy—namely, price stability, the stability of fiscal policy instruments, and sustainable economic growth—requires the interaction of these two policies. This finding supports the first hypothesis.

Chen, Leeper, and Leith [13] used a model in which fiscal and monetary policies are subject to separate targeting rules, formulated by different authorities with distinct objective functions. They also analyzed how the interaction of these policies affects macroeconomic stability. Benigno and Woodford [18] derived optimal monetary and fiscal policy rules by solving the problem of maximizing expected discounted utility in a dynamic stochastic general equilibrium (DSGE) model. Ascari, Florio, and Gobbi [15] examined the interaction between monetary and fiscal policy in a framework where the sole objective of monetary policy was price-level targeting. Cao [11] derived the optimal paths for inflation and taxation in a model with collateral constraints in the banking sector. Svensson [2] analyzed forecast targeting in the context of monetary policy implementation, which allows for the selection of such an interest rate and its growth path that the inflation rate reaches the assumed target.

This article presents selected optimization methods for determining targeting rules, including the linear–quadratic regulator (LQR), Bellman’s dynamic programming, and the Euler’s calculus of variations. The monetary and fiscal targeting rules were derived from a quadratic optimization problem with a finite time horizon (without discounting), an assumed initial condition, and a free terminal condition. The problem was solved subject to a model integrating monetary and fiscal policy as the constraint, which made it possible to determine targeting rules that account for both monetary and fiscal policy objectives. The resulting targeting rules allow for the interaction of both policies to achieve macroeconomic objectives. This study expands and complements existing research and presents empirical results for Poland.

The aim of this article is to compare the solutions obtained from selected optimization methods, such as the linear–quadratic regulator (LQR), Bellman’s dynamic programming, and Euler’s calculus of variations, which are used to derive targeting rules. In this article, we present targeting rules as solutions to an objective function minimization problem, subject to constraints defined by a model that integrates monetary and fiscal policy. The study successfully achieved the research objective formulated in the introduction.

The conclusions are consistent with the evidence and arguments presented. The study successfully compares the optimization methods and demonstrates that they all yield the same optimal decision variables, regardless of the method used. This validates the robustness of the derived targeting rules and confirms that the form of the targeting rules does not depend on the specific optimization technique applied. These conclusions directly address the main question by providing a framework for determining the optimal policy mix for monetary and fiscal policies and highlighting the key policy variables to target

In further research, it would be worthwhile to determine targeting rules, taking into account the case of an infinite time horizon or an assumed terminal condition. Additionally, it would be valuable to solve a stochastic optimal control problem that considers disturbances in the economy. We will also examine the impact of uncertainty in key economic variables, as well as the application of alternative model specifications—such as nonlinear models or models with feedback loops or model with uncertainty and forward-looking expectations—on policy mix rules.

We can also utilize simulating future policy scenarios or exploring trade-offs between inflation control and fiscal sustainability.

A sensitivity analysis of the targeting rules regarding different assumptions or parameters will also be provided.

## 4. Conclusions

The use of optimization problems in monetary and fiscal policy decision-making facilitates the determination of optimal policy rules. Implementing targeting rules allows for continuous policy monitoring and adaptation to new circumstances, which is not feasible with purely instrumental rules. This approach enhances the credibility of macroeconomic policy and supports the effective implementation of these rules. The targeting rules derived in this study provide a comprehensive framework for conducting macroeconomic policy aimed at achieving the primary objectives of both monetary and fiscal policy. The research presented here underscores the benefits of integrating monetary and fiscal policy to attain overarching macroeconomic goals.

Moreover, this study contributes by providing a unified optimization framework that combines both monetary and fiscal policy considerations, which is typically overlooked in the existing literature. The use of multiple optimization methods to solve targeting rules offers a novel comparison of techniques that can be applied to derive policy targets. The inclusion of variables like the interest rate, balance-to-GDP ratio, and debt-to-GDP ratio in the same optimization model adds depth to the subject area and provides a more robust approach to understanding policy interactions.

## Figures and Tables

**Table 1:** Optimal values obtained using the LQR method.

Data	$i_{t}-0.045$	$s_{t}+0.03$	$\pi_{t}-1.025$	$\left(d_{t}-0,55\right)$	$y_{t}-0.031$
2010 Q3	0.0016	0.0000	−0.0030	−0.0070	0.0080
2010 Q4	0.0001	0.0000	−0.0045	0.0073	0.0033
2011 Q1	−0.0003	0.0000	−0.0027	0.0030	0.0015
2011 Q2	−0.0004	0.0000	−0.0019	0.0014	0.0009
2011 Q3	−0.0004	0.0000	−0.0015	0.0008	0.0006
2011 Q4	−0.0003	0.0000	−0.0013	0.0006	0.0005
2012 Q1	−0.0003	0.0000	−0.0011	0.0005	0.0004
2012 Q2	−0.0002	0.0000	−0.0009	0.0004	0.0004
2012 Q3	−0.0002	0.0000	−0.0008	0.0003	0.0003
2012 Q4	−0.0001	0.0000	−0.0007	0.0003	0.0003
2013 Q1	−0.0001	0.0000	−0.0006	0.0002	0.0002
2013 Q2	−0.0001	0.0000	−0.0005	0.0002	0.0002
2013 Q3	−0.0001	0.0000	−0.0004	0.0002	0.0002
2013 Q4	−0.0001	0.0000	−0.0004	0.0002	0.0001
2014 Q1	−0.0001	0.0000	−0.0003	0.0001	0.0001
2014 Q2	−0.0001	0.0000	−0.0003	0.0001	0.0001
2014 Q3	−0.0001	0.0000	−0.0002	0.0001	0.0001
2014 Q4	−0.0001	0.0000	−0.0002	0.0001	0.0001
2015 Q1	0.0000	0.0000	−0.0002	0.0001	0.0001
2015 Q2	0.0000	0.0000	−0.0001	0.0001	0.0001
2015 Q3	0.0000	0.0000	−0.0001	0.0001	0.0000
2015 Q4	0.0000	0.0000	−0.0001	0.0000	0.0000
2016 Q1	0.0000	0.0000	−0.0001	0.0000	0.0000
2016 Q2	0.0000	0.0000	−0.0001	0.0000	0.0000
2016 Q3	0.0000	0.0000	−0.0001	0.0000	0.0000
2016 Q4	0.0000	0.0000	−0.0001	0.0000	0.0000
2017 Q1	0.0000	0.0000	−0.0001	0.0000	0.0000
2017 Q2	0.0000	0.0000	0.0000	0.0000	0.0000
2017 Q3	0.0000	0.0000	0.0000	0.0000	0.0000
2017 Q4	0.0000	0.0000	0.0000	0.0000	0.0000
2018 Q1	0.0000	0.0000	0.0000	0.0000	0.0000
2018 Q2	0.0000	0.0000	0.0000	0.0000	0.0000
2018 Q3	0.0000	0.0000	0.0000	0.0000	0.0000
2018 Q4	0.0000	0.0000	0.0000	0.0000	0.0000
2019 Q1	0.0000	0.0000	0.0000	0.0000	0.0000
2019 Q2	0.0000	0.0000	0.0000	0.0000	0.0000
2019 Q3	0.0000	0.0000	0.0000	0.0000	0.0000
2019 Q4	0.0000	0.0000	0.0000	0.0000	0.0000
2020 Q1	0.0000	0.0000	0.0000	0.0000	0.0000
2020 Q2	0.0000	0.0000	0.0000	0.0000	0.0000
2020 Q3	0.0000	0.0000	0.0000	0.0000	0.0000
2020 Q4	0.0000	0.0000	0.0000	0.0000	0.0000
2021 Q1	0.0000	0.0000	0.0000	0.0000	0.0000
2021 Q2	0.0000	0.0000	0.0000	0.0000	0.0000
2021 Q3	0.0000	0.0000	0.0000	0.0000	0.0000
2021 Q4	0.0000	0.0000	0.0000	0.0000	0.0000
2022 Q1	0.0000	0.0000	0.0000	0.0000	0.0000
2022 Q2	0.0000	0.0000	0.0000	0.0000	0.0000
2022 Q3	0.0000	0.0000	0.0000	0.0000	0.0000
2022 Q4	0.0000	0.0000	0.0000	0.0000	0.0000
2023 Q1	0.0000	0.0000	0.0000	0.0000	0.0000
2023 Q2	0.0000	0.0000	0.0000	0.0000	0.0000
2023 Q3	0.0000	0.0000	0.0000	0.0000	0.0000
2023 Q4	0.0000	0.0000	0.0000	0.0000	0.0000
2024 Q1	0.0000	0.0000	0.0000	0.0000	0.0000
2024 Q2	-	-	0.0000	0.0000	0.0000

Source: Own calculations.

**Table 2:** Optimal values obtained using Bellman’s dynamic programming method.

Data	$i_{t}-0.045$	$s_{t}+0.03$	$\pi_{t}-1.025$	$\left(d_{t}-0,55\right)$	$y_{t}-0.031$
2010 Q3	0.0016	0.0000	−0.0030	−0.0070	0.0080
2010 Q4	0.0001	0.0000	−0.0045	0.0073	0.0033
2011 Q1	−0.0003	0.0000	−0.0027	0.0030	0.0015
2011 Q2	−0.0004	0.0000	−0.0019	0.0014	0.0009
2011 Q3	−0.0004	0.0000	−0.0015	0.0008	0.0006
2011 Q4	−0.0003	0.0000	−0.0013	0.0006	0.0005
2012 Q1	−0.0003	0.0000	−0.0011	0.0005	0.0004
2012 Q2	−0.0002	0.0000	−0.0009	0.0004	0.0004
2012 Q3	−0.0002	0.0000	−0.0008	0.0003	0.0003
2012 Q4	−0.0002	0.0000	−0.0007	0.0003	0.0003
2013 Q1	−0.0001	0.0000	−0.0006	0.0002	0.0002
2013 Q2	−0.0001	0.0000	−0.0005	0.0002	0.0002
2013 Q3	−0.0001	0.0000	−0.0004	0.0002	0.0002
2013 Q4	−0.0001	0.0000	−0.0004	0.0002	0.0001
2014 Q1	−0.0001	0.0000	−0.0003	0.0001	0.0001
2014 Q2	−0.0001	0.0000	−0.0003	0.0001	0.0001
2014 Q3	−0.0001	0.0000	−0.0002	0.0001	0.0001
2014 Q4	−0.0001	0.0000	−0.0002	0.0001	0.0001
2015 Q1	0.0000	0.0000	−0.0002	0.0001	0.0001
2015 Q2	0.0000	0.0000	−0.0001	0.0001	0.0001
2015 Q3	0.0000	0.0000	−0.0001	0.0001	0.0000
2015 Q4	0.0000	0.0000	−0.0001	0.0000	0.0000
2016 Q1	0.0000	0.0000	−0.0001	0.0000	0.0000
2016 Q2	0.0000	0.0000	−0.0001	0.0000	0.0000
2016 Q3	0.0000	0.0000	−0.0001	0.0000	0.0000
2016 Q4	0.0000	0.0000	−0.0001	0.0000	0.0000
2017 Q1	0.0000	0.0000	−0.0001	0.0000	0.0000
2017 Q2	0.0000	0.0000	0.0000	0.0000	0.0000
2017 Q3	0.0000	0.0000	0.0000	0.0000	0.0000
2017 Q4	0.0000	0.0000	0.0000	0.0000	0.0000
2018 Q1	0.0000	0.0000	0.0000	0.0000	0.0000
2018 Q2	0.0000	0.0000	0.0000	0.0000	0.0000
2018 Q3	0.0000	0.0000	0.0000	0.0000	0.0000
2018 Q4	0.0000	0.0000	0.0000	0.0000	0.0000
2019 Q1	0.0000	0.0000	0.0000	0.0000	0.0000
2019 Q2	0.0000	0.0000	0.0000	0.0000	0.0000
2019 Q3	0.0000	0.0000	0.0000	0.0000	0.0000
2019 Q4	0.0000	0.0000	0.0000	0.0000	0.0000
2020 Q1	0.0000	0.0000	0.0000	0.0000	0.0000
2020 Q2	0.0000	0.0000	0.0000	0.0000	0.0000
2020 Q3	0.0000	0.0000	0.0000	0.0000	0.0000
2020 Q4	0.0000	0.0000	0.0000	0.0000	0.0000
2021 Q1	0.0000	0.0000	0.0000	0.0000	0.0000
2021 Q2	0.0000	0.0000	0.0000	0.0000	0.0000
2021 Q3	0.0000	0.0000	0.0000	0.0000	0.0000
2021 Q4	0.0000	0.0000	0.0000	0.0000	0.0000
2022 Q1	0.0000	0.0000	0.0000	0.0000	0.0000
2022 Q2	0.0000	0.0000	0.0000	0.0000	0.0000
2022 Q3	0.0000	0.0000	0.0000	0.0000	0.0000
2022 Q4	0.0000	0.0000	0.0000	0.0000	0.0000
2023 Q1	0.0000	0.0000	0.0000	0.0000	0.0000
2023 Q2	0.0000	0.0000	0.0000	0.0000	0.0000
2023 Q3	0.0000	0.0000	0.0000	0.0000	0.0000
2023 Q4	0.0000	0.0000	0.0000	0.0000	0.0000
2024 Q1	0.0000	0.0000	0.0000	0.0000	0.0000
2024 Q2	-	-	0.0000	0.0000	0.0000

Source: Own calculations.

**Table 3:** Optimal values obtained using the discrete Euler calculus of variations.

Data	$i_{t}-0.045$	$s_{t}+0.03$	$\pi_{t}-1.025$	$\left(d_{t}-0,55\right)$	$y_{t}-0.031$
2010 Q3	0.0015	0.0000	−0.0030	−0.0070	0.0080
2010 Q4	0.0005	0.0000	−0.0045	0.0073	0.0034
2011 Q1	0.0001	0.0000	−0.0027	0.0031	0.0013
2011 Q2	0.0000	0.0000	−0.0020	0.0011	0.0004
2011 Q3	−0.0001	0.0000	−0.0016	0.0004	0.0001
2011 Q4	−0.0001	0.0000	−0.0015	0.0001	0.0000
2012 Q1	−0.0001	0.0000	−0.0014	0.0000	−0.0001
2012 Q2	−0.0001	0.0000	−0.0013	−0.0001	−0.0001
2012 Q3	−0.0001	0.0000	−0.0013	−0.0001	−0.0001
2012 Q4	−0.0001	0.0000	−0.0013	−0.0001	−0.0001
2013 Q1	−0.0001	0.0000	−0.0012	−0.0001	−0.0001
2013 Q2	−0.0001	0.0000	−0.0012	−0.0001	−0.0001
2013 Q3	−0.0001	0.0000	−0.0012	−0.0001	−0.0001
2013 Q4	−0.0001	0.0000	−0.0011	−0.0001	−0.0001
2014 Q1	−0.0001	0.0000	−0.0011	−0.0001	−0.0001
2014 Q2	−0.0001	0.0000	−0.0011	−0.0001	−0.0001
2014 Q3	−0.0001	0.0000	−0.0010	−0.0001	−0.0001
2014 Q4	−0.0001	0.0000	−0.0010	−0.0001	−0.0001
2015 Q1	−0.0001	0.0000	−0.0010	−0.0001	−0.0001
2015 Q2	−0.0001	0.0000	−0.0010	−0.0001	−0.0001
2015 Q3	−0.0001	0.0000	−0.0009	−0.0001	−0.0001
2015 Q4	−0.0001	0.0000	−0.0009	−0.0001	−0.0001
2016 Q1	−0.0001	0.0000	−0.0009	−0.0001	−0.0001
2016 Q2	−0.0001	0.0000	−0.0009	−0.0001	−0.0001
2016 Q3	−0.0001	0.0000	−0.0008	−0.0001	−0.0001
2016 Q4	−0.0001	0.0000	−0.0008	−0.0001	−0.0001
2017 Q1	0.0000	0.0000	−0.0008	−0.0001	−0.0001
2017 Q2	0.0000	0.0000	−0.0008	−0.0001	−0.0001
2017 Q3	0.0000	0.0000	−0.0008	−0.0001	−0.0001
2017 Q4	0.0000	0.0000	−0.0007	0.0000	−0.0001
2018 Q1	0.0000	0.0000	−0.0007	0.0000	−0.0001
2018 Q2	0.0000	0.0000	−0.0007	0.0000	−0.0001
2018 Q3	0.0000	0.0000	−0.0007	0.0000	0.0000
2018 Q4	0.0000	0.0000	−0.0007	0.0000	0.0000
2019 Q1	0.0000	0.0000	−0.0006	0.0000	0.0000
2019 Q2	0.0000	0.0000	−0.0006	0.0000	0.0000
2019 Q3	0.0000	0.0000	−0.0006	0.0000	0.0000
2019 Q4	0.0000	0.0000	−0.0006	0.0000	0.0000
2020 Q1	0.0000	0.0000	−0.0006	0.0000	0.0000
2020 Q2	0.0000	0.0000	−0.0006	0.0000	0.0000
2020 Q3	0.0000	0.0000	−0.0005	0.0000	0.0000
2020 Q4	0.0000	0.0000	−0.0005	0.0000	0.0000
2021 Q1	0.0000	0.0000	−0.0005	0.0000	0.0000
2021 Q2	0.0000	0.0000	−0.0005	0.0000	0.0000
2021 Q3	0.0000	0.0000	−0.0005	0.0000	0.0000
2021 Q4	0.0000	0.0000	−0.0005	0.0000	0.0000
2022 Q1	0.0000	0.0000	−0.0005	0.0000	0.0000
2022 Q2	0.0000	0.0000	−0.0005	0.0000	0.0000
2022 Q3	0.0000	0.0000	−0.0004	0.0000	0.0000
2022 Q4	0.0000	0.0000	−0.0004	0.0000	0.0000
2023 Q1	0.0000	0.0000	−0.0004	0.0000	0.0000
2023 Q2	0.0000	0.0000	−0.0004	0.0000	0.0000
2023 Q3	0.0000	0.0000	−0.0004	0.0000	0.0000
2023 Q4	0.0000	0.0000	−0.0004	0.0000	0.0000
2024 Q1	0.0000	0.0000	−0.0004	0.0000	0.0000
2024 Q2	-	-	−0.0004	0.0000	0.0000

Source: Own calculations.

## Data Availability

The links to the datasets are provided in the article.

## Appendix A

% Problem parameters

T = 57; % Time horizon

n = 3; % Number of state variables

m = 2; % Number of control variables

A = [0.9612212, 0.222054, 0; 0, 0, 0.9102691; 0.072456, 0, 0.5819569];

B = [−0.0431, 0; 0, 0.0070764; −0.17166, 0];

Q = [1/3, 0, 0; 0, 1/3, 0; 0, 0, 1/3];

R = [0.5, 0; 0, 0.5];

% Initialization of cost and control matrices

V = zeros(n, n, T+1); % Minimum cost

K = zeros(m, n, T); % Optimal controls

% Terminal condition

V(:, :, T+1) = Q;

% Dynamic programming algorithm

for t = T:−1:1

% Bellman equation

H = R + B’ * V(:, :, t+1) * B; % Hessian of the cost function

G = B’ * V(:, :, t+1) * A; % Gradient of the cost function

K(:, :, t) = -inv(H) * G; % Optimal control

V(:, :, t) = Q + A’ * V(:, :, t+1) * (A + B * K(:, :, t)); % Minimum cost

end

% Display results

disp(‘Optimal controls K:‘);

disp(K);

% Initial state

x0 = [−0.003; −0.007; −0.008]; % Initial state

x = zeros(n, T+1);

u = zeros(m, T);

x(:, 1) = x0;

for t = 1:T

u(:, t) = K(:, :, t) * x(:, t); % Control at time t

x(:, t+1) = A * x(:, t) + B * u(:, t); % State update

end

% Display state trajectory

disp(‘State trajectory:‘);

disp(x);

% Display control trajectory

disp(‘Control trajectory:‘);

disp(u);
